# A Mechanically Transmitted DNA Mycovirus Is Targeted by the Defence Machinery of Its Host, *Botrytis cinerea*

**DOI:** 10.3390/v13071315

**Published:** 2021-07-07

**Authors:** Mahmoud E. Khalifa, Robin M. MacDiarmid

**Affiliations:** 1 The New Zealand Institute for Plant and Food Research Limited, Auckland 1025, New Zealand; Robin.MacDiarmid@plantandfood.co.nz; 2 Botany and Microbiology Department, Faculty of Science, Damietta University, Damietta 34517, Egypt; 3 School of Biological Sciences, The University of Auckland, Auckland 1010, New Zealand

**Keywords:** *Botrytis cinerea*, mycovirus, ssDNA, *Genomoviridae*, *Gemydayirivirus*, hypovirulence, siRNA

## Abstract

Eukaryotic circular single-stranded DNA (ssDNA) viruses were known only to infect plants and vertebrates until the discovery of the isolated DNA mycovirus from the fungus *Sclerotinia sclerotiorum*. Similar viral sequences were reported from several other sources and classified in ten genera within the *Genomoviridae* family. The current study reports two circular ssDNA mycoviruses isolated from the phytopathogen *Botrytis cinerea*, and their assignment to a newly created genus tentatively named *Gemydayirivirus*. The mycoviruses, tentatively named botrytis gemydayirivirus 1 (BGDaV1) and BGDaV2, are 1701 and 1693 nt long and encode three and two open reading frames (ORFs), respectively. Of the predicted ORFs, only ORF I, which codes for a replication initiation protein (Rep), shared identity with other proteins in GenBank. BGDaV1 is infective as cell-free purified particles and confers hypovirulence on its natural host. Investigation revealed that BGDaV1 is a target for RNA silencing and genomic DNA methylation, keeping the virus at very low titre. The discovery of BGDaV1 expands our knowledge of the diversity of genomoviruses and their interaction with fungal hosts.

## 1. Introduction

*Botrytis cinerea*, unlike the majority of other *Botrytis* species that are restricted to certain hosts, is a ubiquitous ascomycetous phytopathogen capable of infecting more than 1400 host species worldwide [1]. This fungus constrains the production of a wide range of fruit and flower crops, such as grapes and cyclamens, in which it causes several pre- and post-harvest diseases including grey mould, leaf blight, blossom blight, bunch rot disease, and post-harvest fruit rots [2], with grey mould being the most common. Diseases caused by *B. cinerea* are commonly controlled through the application of chemical fungicides. However, this use of chemical fungicides is of increasing concern because of their hazardous impact on the environment, potential effects on human health, and the ability of host fungi to develop resistance to fungicides [3]. These concerns, along with continuing loss of certain fungicides in the market, are the driving force behind the motivation to evaluate other means of fungal disease control.

Fungal viruses (mycoviruses) have been reported in various phytopathogenic fungi [4] from all four phyla of true fungi [3]. Hypovirulence-associated mycoviruses (those that reduce the pathogenicity of their fungal host) have been exploited as biological control agents for fungal pathogens; however, because of the inability to transmit these mycoviruses easily between vegetatively incompatible groups [5,6] it has been difficult to develop commercial mycovirus biocontrol tools for these phytopathogenic fungi. The biology of three DNA mycoviruses has been described. Of the three DNA mycoviruses, Sclerotinia sclerotiorum hypovirulence-associated DNA virus 1 (SsHADV-1) [7], currently named sclerotinia gemycircularvirus 1 (SGCV1) [8], and Fusarium graminearum gemytripvirus 1 (FgGMTV1) [9] were hypovirulence-associated. Until recently, all attempts have failed to infect fungal mycelia extracellularly (mechanically transmit mycoviruses) with purified mycoviruses in the presence of a fungal cell wall [4]. Although this fact remains valid for RNA mycoviruses, it was clearly demonstrated that purified particles of SGCV1 are infectious when applied to *Sclerotinia sclerotiorum* mycelia in vitro or when applied to plant leaves prior to or post infection by *S. sclerotiorum* [10].

Levels of mycovirus accumulation with fungal hosts are determined by the efficiency of the host’s defence machinery on one hand and the counter-defence used by mycoviruses to combat antiviral activity [11]. While RNA silencing and DNA methylation have been reported as efficient antiviral defence systems in several fungal and/or plant hosts, some viruses have the ability to block these defence systems by producing viral suppressors of RNAi (VSRs), leading to virus accumulation [12]. In this research we investigated the possibility of mechanically transmitted mycoviruses being present in *B. cinerea*, a fungus within a genus closely related to *Sclerotinia*.

## 2. Materials and Methods

### 2.1. Fungal Isolates and Culturing Conditions

Five hundred isolates of *B. cinerea* were obtained from Manaaki Whenua—Landcare Research, New Zealand. These isolates were chosen on the basis that they were isolated from asymptomatic plants, thereby increasing the chances of finding hypovirulent isolates and/or circular DNA mycoviruses. Isolate 702 is a virus-free recipient isolate used in transmission experiments. All isolates were subcultured and maintained on potato dextrose agar (PDA) at 4 °C until used.

### 2.2. Viral Nucleic Acid Purification, Enrichment and Sequencing

Isolates of *B. cinerea* were cultured on cellophane-covered PDA and incubated at 20 °C for 5 days. Approximately 250 mg of each isolate mycelium was collected and mycelia combined in groups of ten (resulting in 50 samples) prior to virus-like particle (VLP) partial purification and DNA extraction using a modified method of Rosario et al. [13]. Fungal mycelia were homogenised and mixed with 5 mL of SM (0.1 M NaCl, 50 mM Tris-HCl (pH 7.4), 10 mM MgSO_4_). Homogenates were clarified by centrifugation at 10,000× *g* for 5 min and supernatants were filtered through 0.45-µm syringe filters. Total viral nucleic acid was extracted from these filtrates using a High Pure Viral Nucleic Acid Large Volume Kit (Roche) according to the manufacturer’s protocol and enriched for circular DNA by rolling-circle amplification (RCA) using the Illustra™ TempliPhi™ DNA Amplification Kit (GE Healthcare) as described by the manufacturer. RCA products from the 50 samples (representing the 500 isolates) were equimolar pooled before proceeding to sequencing using an Illumina Hiseq2000 100 bp at Macrogen Inc. (Seoul, South Korea).

### 2.3. Bioinformatics and Illumina Sequencing Analysis

Illumina reads with quality scores of less than Q20 were filtered out using the Galaxy Project server [14], and the remaining reads were trimmed to remove low quality sequence stretches at the 5′ end as determined by the FastQC report (http://www.bioinformatics.babraham.ac.uk/projects/fastqc/) (accessed on 30 January 2015). This was followed by assembling the reads into contigs using the de novo assembly tool of Geneious R8.1 [15] set to medium sensitivity and default parameters. Consensus sequences of assembled contigs longer than 1 kb were used to identify circular virus-like sequences using BLASTx analysis against the non-redundant (nr) database of NCBI.

### 2.4. Detection and Sequence Confirmation of Botrytis Gemydayirivirus 1 (BGDaV1)

Two pairs of primers (P01-1F1 5′ GGAGATACAAGCCAAAGGGG 3′ + P01-1R1 5′ CTGTTTGCGCCTCTTTGGGG 3′ and P01-1F2 5′ CTACTCTTCATTTGCTGCTGCC 3′ + P01-1R2 5′ CTTGCCCAACGACCTAGCCC 3′) were designed for PCR detection and amplification of two overlapping segments that cover the full-length sequence of the circular viral genomes of this study. The 50 presumed viral DNA pools (no RCA enrichment) were PCR-screened for the presence of BGDaV1 sequence. DNA was purified from isolates of each pool that tested positive for BGDaV1 using a ZR Fungal/Bacterial DNA MiniPrep or a High Pure Viral Nucleic Acid Kit (Roche). Purified DNA was used for PCR-screening and sequencing of BGDaV1.

### 2.5. BGDaV1 and BGDaV2 Sequence and Phylogenetic Analyses

Circular sequences were assembled using Geneious R8.1 [15]. Multiple sequence alignment and detection of conserved motifs of the replication initiation proteins (Reps) of the BGDaV1 and BGDaV2 was carried out using MUSCLE [16] and illustrated using Weblogo3 [17]. For phylogenetic analysis, Rep amino acid (aa) sequences of circular ssDNA viruses were aligned using PROMALS3D [18], and columns containing gaps were removed from the alignment using trimAl v1.3 set to strict mode [19]. The best fitting-model was selected and maximum likelihood tree inferred using PhyML 3.0 [20]. Branches with <75% SH-like branch support were collapsed using TreeGraph 2 software [21].

### 2.6. BGDaV1 Particles Purification and Associated DNA

A 10- to 20-g portion of mycelia was ground to a fine powder in liquid nitrogen using a sterilised mortar and pestle. The powder was transferred to a sterilised 50-mL Falcon tube and a 20-mL aliquot of 0.1 M sodium phosphate buffer (pH 7) was added. The tube was shaken on ice for 10 min, a 10-mL aliquot of chloroform was added and the tube was further shaken on ice for 30 min prior to being centrifuged at 10,000× *g* for 30 min at 4 °C. The aqueous phase was separated between two ultracentrifuge tubes and the tubes were spun at 120,000× *g* for 80 min. Following ultracentrifugation, the pellet was resuspended in a small volume of 0.02 M sodium phosphate buffer (pH 7), the suspension was clarified by low-speed centrifugation at 10,000× *g* for 10 min at 4 °C, the supernatant was made up to 10 mL using 0.02 M sodium phosphate buffer (pH 7) and ultracentrifugation repeated as above. The resultant pellet was resuspended and clarified as above and the supernatant was examined by transmission electron microscope for the presence of virus particles. Particles-associated DNA was purified using a High Pure Viral Nucleic Acid Kit (Roche).

### 2.7. Screening BGDaV1-Containing Isolates for the Presence of RNA Viruses

A dsRNA purification protocol, as described by Khalifa and Pearson [22], as well as particle purification and electron microscopy, were used to screen BGDaV1-containing isolates for the presence of RNA viruses. The purified dsRNAs were electrophoretically separated on a SYBR safe pre-stained 1% (*w*/*v*) agarose gel in 1× TAE buffer (pH 7.4), visualised and photographed under UV using a Gel Doc (Bio-Rad, Hercules, CA, USA).

### 2.8. Infectivity of BGDaV1 as Purified Particles

To study the mechanical transmissibility of BGDaV1, purified particles associated with isolates 339-13, 339-49 and 339-101 were applied to the growing margins of a virus-free *B. cinerea* isolate 702. After a 4-day incubation period at 20 °C, mycelial plugs were transferred from the growing margins of the inoculated colonies onto new PDA plates to produce isolates 702-V13, 702-V49 and 702-V101. Total DNA was extracted from the resultant isolates and successful transmission of BGDaV1 was tested by PCR using primers P01-1F1 and P01-1R1. The transmission experiment was repeated three times, and PCR testing of BGDaV1 was performed on three successive subcultures of the new progeny. Positive DNA extraction control was performed by amplifying the non-coding internal transcribed spacer (ITS) regions using the primer pair ITS4/ITS5 [23].

### 2.9. Effect of BGDaV1 Infection on B. cinerea

To study the effect of BGDaV1 on the growth rate of *B. cinerea*, PBS and BGDaV1 purified particles in PBS were applied to the growing margins of isolate 702. Following incubation at 20 °C, mycelia growth rate was measured for the PBS-challenged and particles-challenged sides. A similar comparison was made using subcultures of the developed colonies. Each treatment was performed in triplicate. For virulence assay, mycelial plugs from cultures produced from the transmission experiment were applied to detached canola leaves. Also, a mixture of purified particles from isolates 339-49 and 339-101 was applied directly to canola leaves, resulting in prophylactic application of the virus, and a mycelial plug of the virus-free isolate 702 was applied to the virus mixture on canola leaves. Inoculated leaves were incubated for 4–5 days before the diameter of the *B. cinerea* lesion on the leaf was measured. Each treatment was performed in triplicate.

### 2.10. Small Interfering RNAs (siRNA) and Methylation

The siRNA fraction was purified from BGDaV1-infected *B. cinerea* isolates using the mirPremier™ microRNA Isolation Kit, then processed to generate small RNA (sRNA) libraries for Illumina sequencing at Macrogen Inc. (Seoul, South Korea). Illumina reads generated were mapped using Geneious against the BGDaV1 genome to identify siRNAs originating from that virus or mapped against the host genome to determine the virus-siRNA to host-siRNA ratio.

DNA was purified from BGDaV1 infected isolates and subjected to bisulfite conversion using a bisulfite conversion kit (Qiagen) prior to RCA, which preferably amplifies circular genomes (i.e., that of BGDaV1) as well as a small fraction of the host DNA (*B. cinerea*). RCA products were fragmented and sequenced using Illumina Hiseq2500 100 bp at Macrogen Inc. (Seoul, South Korea). Unmethylated cytosines were identified as thymines instead of the encoded cytosine. Methylation rates were also determined for an internal control, the ITS region (accession number: KP151604) of *B. cinerea* [24].

### 2.11. Data Analysis

Statistical analysis of the data was performed using one-way ANOVA with Tukey’s multiple comparisons test using STATISTICA software.

## 3. Results and Discussion

### 3.1. Circular Rep-Encoding ssDNAs (CRESS-DNAs) of B. cinerea

Analysis of Illumina short reads from 500 *B. cinerea* isolates revealed the presence of at least two *B. cinerea* extra-chromosomal DNA virus-like sequences (Sequence A and B) with encoded proteins similar to those of circular-genome-containing fungal and plant DNA viruses, including SGCV1 as determined through BLASTx searches of the NCBI databases.

### 3.2. Sequence and Phylogenetic Analyses of the Extra-Chromosomal DNA Elements Associated with B. cinerea

**Figure 1:**
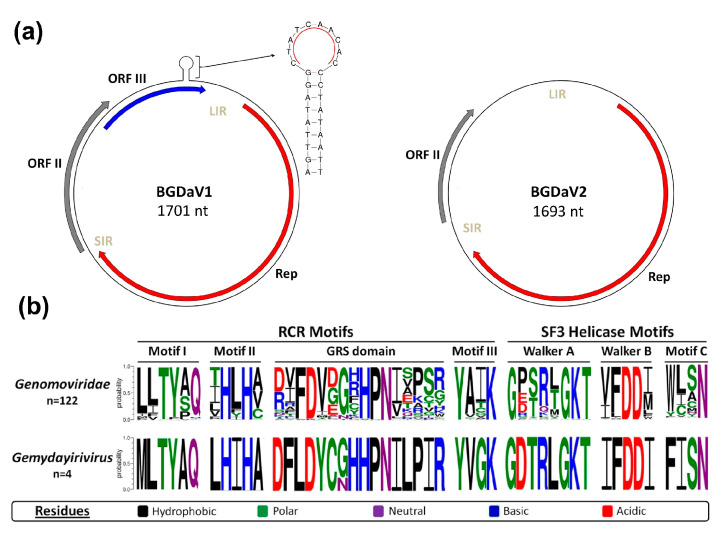
(**a**) Schematic illustration of the genome organisation of BGDaV1 and BGDaV2; (**b**) Rep aa sequence motifs of BGDaV1 and BGDaV2 (*Gemydayirivirus*) and of the family *Genomoviridae* as illustrated by WebLogo3.

CRESS-DNA viruses are characterised by small genomes (∼1.7–3 kb) that encode at least two proteins: a Rep and a capsid protein (Cap) [25]. The Sanger sequencing confirmed that the sequence of the first *B. cinerea* extra-chromosomal DNA element (Sequence A) (Figure 1a) is 1701 nucleotides (nt) with three unidirectional open reading frames (ORFs). The longest ORF (ORF I) is 966 nt (nt position: 152-1117), whereas the remaining two ORFs, ORF II and III, are overlapping, with lengths of 375 (nt position: 1137–1511) and 294 nt (nt position: 1454-46), respectively. The virus-like genome contains two intergenic regions: a long intergenic region (LIR) of 105 nt (nt position: 74–151) between ORFs III and ORF I, and a short intergenic region (SIR) of 19 nt (nt position: 1118–1136) between ORFs I and II. ORF I codes a 321 aa protein with a calculated molecular mass of 36.7 kDa. ORFs II and III code for 124 and 97 aa proteins with calculated molecular masses of ~14 and 10.4 kDa, respectively. A conserved nonanucleotide motif is required for rolling-circle replication (RCR) of CRESS-DNA viruses [13]. A putative nonanucleotide sequence motif (CTATCAACAC) was identified at the top of a stem-loop structure located at the terminus of ORF III.

Sequence B is 1693 nt long and encodes two ORFs that are 966 (ORF I) and 282 nt (ORF II) in length (Figure 1a). The two ORFs are separated by LIR and SIR of 350 and 95 nts, respectively. ORF I and II have the potential to encode proteins of 321 and 93 aa with calculated molecular weights of ~36.7 and 10.6 kDa, respectively. The exact position of a putative nonanucleotide sequence motif could not be confirmed. Possible positions and sequences of the putative nonanucleotide motifs include CTATAACATC (current nt position: 1–10) as compared to that of Sequence A (CTATCAACAC), TAACAGTACC as compared to the putative nonanucleotide motif sequence of Botrytis cinerea genomovirus 1 (BcGV1-LD17-26) (TAACAGTACC; nt position: 113–122) [26] or TAATTAATTC as compared to the putative nonanucleotide motif sequence of Botrytis cinerea ssDNA virus 1 (BcSDV1-BCS11_DN160) (TAATTAATTC; nt position: 1132–1141) [27].

A BLASTp search of the translated ORF I sequence against the NCBI database revealed that Sequence A and B share the highest aa sequence identities with BcGV1-LD17-26 (91.9 and 99.38%) and BcSDV1-BCS11_DN160 (91.9 and 98.75%), respectively [26,27]. These proteins also had ~39 and ~40% similarity with Reps of black robin-associated gemykibivirus 1 (nt sequence accession no: KF371634) detected in New Zealand [28] and dragonfly-associated gemykibivirus 1 (nt sequence accession no: JX185430) identified in USA [29], respectively. They also shared similarities with Reps of other circular ssDNA viruses from various sources. The high aa sequence identities between sequence A and BcGV1-LD17-26 as well as between Sequence B and BcSDV1-BCS11_DN160 suggest that they can be strains of the same virus, respectively.

Searching the NCBI conserved domain database (CDD) showed that these putative Reps had two conserved domains: geminivirus Rep catalytic domain (Gemini_AL1 superfamily; pfam00799), and geminivirus Rep protein central domain (Gemini_AL1_M superfamily; pfam08283), with conserved motifs for RCR: (Motif I (MLTYAQ), Motif II (LHIHA), GRS (Sequence A: DFLDYCNHHPNILPIR and Sequence B: DFLDYCGHHPNILPIR) and Motif III (YVGK)) and SF3 Helicase (Walker-A (GDTRLGKT), Walker-B (IFDDI) and Motif C (FISN)) motifs described for ssDNA viruses (Figure 1b).

**Figure 2:**
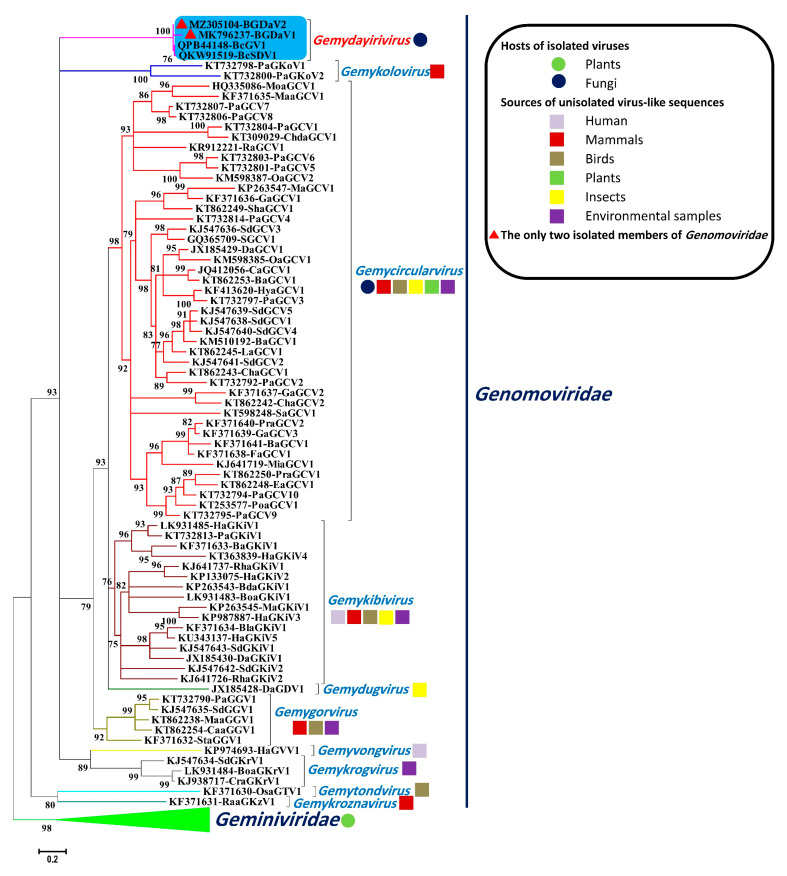
Phylogenetic relationship between the Reps of BGDaV1, BGDaV2 and other selected circular ssDNA viruses including genomoviruses with monopartite genome and members of *Geminiviridae* family. The maximum likelihood tree was inferred using PhyML 3.0 [20] with the LG + G + I + F as the best evolutionary model. The SH-like support values are indicated by numbers on the branches. Branches with <75% SH-like branch support have been collapsed using TreeGraph 2 software [21].

The maximum likelihood (ML) tree based on the Rep sequences of *B. cinerea* extra-chromosomal DNA elements and other selected circular ssDNA sequences (Figure 2) revealed that Sequence A and B were closely related to, but distinct from, sequences of *Genomoviridae*. Using the taxonomic framework for the classification of genomoviruses based on the sequence information [8] and as suggested by the ML phylogenetic analysis, the extra-chromosomal DNA elements identified in this study probably represent a novel viral species of a previously unrecognised genus in the family *Genomoviridae* as recently suggested for BcGV1 [26]. Following the basis of the nomenclature of *Genomoviridae* genera [8], the name *Gemydayirivirus* was proposed for the newly created genus, to accommodate the *B. cinerea* extra-chromosomal DNA elements of this study, which we tentatively named BGDaV1 (Sequence A) and BGDaV2 (Sequence B). As for the nine recognised genera of *Genomoviridae* with monopartite genomes, the novel genus name is an acronym of words geminivirus-like and myco-like dayiri virus (dayiri means circular in Maṣri; Egyptian Arabic). The recently described *Genomoviridae* virus family currently includes ten genera, of which *Gemycircularvirus* and *Gemytripvirus* accommodate the only isolated ssDNA mycovirus: SGCV1 [8]. In the era of next-generation sequencing, which made virus discovery more feasible, the family has grown rapidly to include several genomes belonging to 237 ICTV-approved species associated with different sources such as plant materials [30], mosquitoes [31], faecal matter [28,32], sewage samples [33], water samples [34], and human blood and serum samples [35,36].

The Cap proteins of a virus together assemble to encapsidate the viral genome and are often highly diverse among virus families, but in most cases, Caps encoded by divergent CRESS-DNA viruses share no homology [33]. Putative Cap-encoding genes were not identifiable in the BGDaV1 sequence, as neither ORF II (Sequence A and B) nor III (Sequence A) shared any similarities with known CRESS DNA virus Caps apart from the hypothetical proteins encoded by ORF II of BcGV1 [26], which shares ~93% aa identity with the proteins encoded by ORF II of BGDaV1 and BGDaV2. As for BGDaV1, CRESS DNA viruses with two unknown ORFs and no detectable putative Cap genes have been previously reported, such as McMurdo Ice Shelf pond-associated circular DNA virus-4 and -8 [37] and sewage-associated circular DNA virus-31 [33]. Moreover, genome architectures that are almost similar to that of BGDaV1 have been previously reported [33]. Viruses with a size similar to BGDaV1 such as members of *Nanovirus* genus have short Cap genes. An example is subterranean clover stunt virus, which has a 169 aa long Cap [38].

Multiple Cap genes have been reported for some viruses, such as members of the RNA virus family *Closteroviridae* and genera *Sadwavirus* and *Comovirus*. However, the genomes of such RNA viruses are almost 10 times larger than the DNA genome of BGDaV1 and can afford to have multiple Cap genes. It has been recently reported, for the first time, that CRESS genome CPs are encoded by two divergent gene homologs [39]. Whether or not BGDaV1 ORFs II and III produce the Cap is not known. Moreover, a number of unusual translational mechanisms for the expression of extended viral genes, such as leaky scanning in overlapping ORFs, stop codon read through and ribosomal frameshifting, are utilised by several RNA viruses [40,41]. In this context, it is also not known if BGDaV1 uses similar translational strategies. Although mass spectroscopic analysis of BGDaV1 Cap was not performed in the current study because of the low purified virus yield, which was also the case for BcGV1 [26], this is a future research priority. Since ORF III is absent in BcGV1 [26], BcSDV1 (MN625247) and BGDaV2 of this study, it is more probable that BGDaV1 and BGDaV2 Cap is encoded for by ORF II; and ORF III may have an unknown function. As BGDaV1 is unique and less closely related to *Botrytis* DNA viruses than BGDaV2, we focused on BGDaV1.

### 3.3. BGDaV1 Particles and DNA

**Figure 3:**
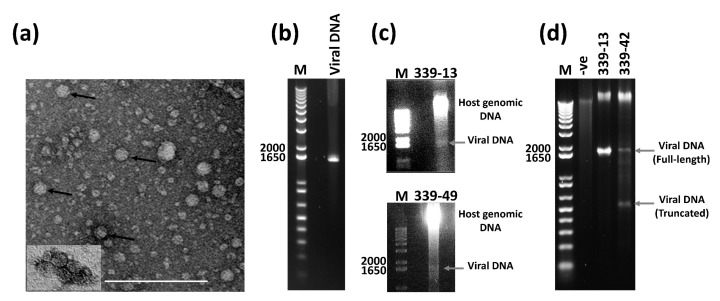
(**a**) Virus particles purified from isolate 339-13 as observed under transmission electron microscopy (TEM); Bar = 200 nm; (**b**) DNA isolated from BGDaV1 purified particles; (**c**) Total DNA extracted from mycelia of two BGDaV1-containing isolates; (**d**) *Eco*RV-digested RCA products of circular DNA associated with isolates 339-13 and 339-42. M: 1kb Plus DNA molecular weight marker (Invitrogen).

To assign BGDaV1 to a specific isolate from the 500 *B. cinerea* isolates used in this study, PCR testing and Sanger sequencing revealed that only eleven of them (isolates # 339-13, 339-19, 339-30, 339-34, 339-38, 339-42, 339-48, 339-49, 339-98, 339-99 and 339-101) contained BGDaV1, all of which were isolated from a single New Zealand vineyard. A few isometric virus particles (~20 nm in diameter; Figure 3a) were observed in the purified preparation from BGDaV1-containing *B. cinerea* isolates using transmission electron microscopy (TEM). Virus particles with similar morphology were previously observed for SGCV1 [7] and BcGV1 [26]. Purified particles were DNase-treated then used to purify their associated nucleic acid. Agarose gel electrophoresis of this purified nucleic acid revealed the presence of ~1.6 kb DNA fragment (Figure 3b), indicating that the BGDaV1 DNA element is encapsidated. Attempts to co-purify the BGDaV1 DNA along with the fungal host genome (total fungal DNA) from different isolates, followed by its detection by agarose gel electrophoresis, failed until more than ~50 µg of total DNA was loaded on the gel (Figure 3c). However, PCR successfully detected BGDaV1 in the same total fungal DNA preparations. These results are probably explained by the viral DNA being present in a low concentration that is not easily detectable by agarose gel electrophoresis, and this is consistent with the observation of a few particles by TEM as also reported for BcGV1 [26]. RNA viruses that were similarly undetectable using electrophoresis because of low virus titres have been previously reported [42]. Moreover, it was also possible to detect the linear dsDNA form of BGDaV1 by using RCA to enrich for the viral circular DNA, followed by digestion of the RCA product using a restriction enzyme that cleaved the amplified BGDaV1 genome only once (Figure 3d). This RCA also revealed the presence of a defective form (truncated genome) of BGDaV1 (~500 nt) in one isolate 339-42 (Figure 3d), similar to that detected for SGCV1 [7]. BGDaV1 DNA isolated from viral particles migrated faster than that purified from the fungal mycelia and the product amplified by RCA. This was not unexpected, because of the single-stranded nature of the DNA elements accommodated by viral particles in comparison with the dsDNA forms obtained by RCA and that of the replicative form of BGDaV1 in the host mycelia.

### 3.4. Presence of RNA Viruses in BGDaV1-Containing Isolates

Mixed mycovirus infection of several fungal hosts has been reported [42,43,44]. Therefore, BGDaV1-containing isolates were tested for the presence of (+)ssRNA and dsRNA viruses using a dsRNA detection method and for (−)ssRNA viruses using TEM. As shown in Supplementary Materials, Figure S1, dsRNAs were detected in seven isolates. Isolates 339-13, 339-49, 339-99 and 339-101 appeared to be dsRNA free, of which isolates 339-13, 339-49 and 339-101 appeared to be devoid of other viral particles that may represent (−)ssRNA viruses and hence suitable for further transmission and pathogenicity experiments.

### 3.5. Mechanical Transmissibility and Stability of BGDaV1 Purified Particles

**Figure 4:**
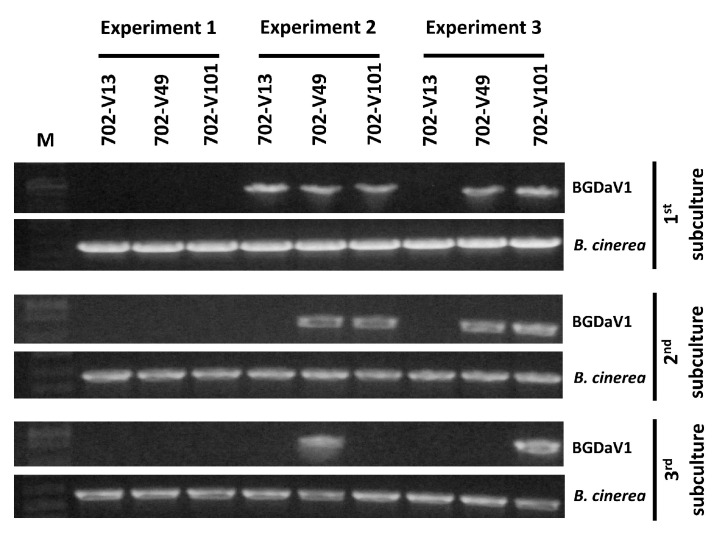
Detection of BGDaV1 in three separate transmission trials. BGDaV1 was detected using PCR with primers P01-1F1 and P01-1R1 (830 bp amplicon; BGDaV1) on total fungal DNA extracts acquired from three successive subcultures of each treatment. Positive DNA extraction control was performed by amplifying the ITS regions of B. cinerea genome. M: 1kb Plus DNA Ladder (Invitrogen).

The use of mycoviruses as effective biological control agents is challenging because of the lack of an extracellular phase in their transmission cycle [45]; the exception is the ssDNA mycovirus (SGCV1), which is known to be infectious in fungi as purified particles [10]. The infectivity of BGDaV1 purified particles was tested three times in separate transmission experiments. Following application of cell-free purified particles to the growing margins of a virus-free *B. cinerea* isolate 702, DNA extracts from recovered colonies (702-V13, 702-V49 and 702-V101) were PCR-tested for the presence of BGDaV1 DNA using primers P01-1F1 and P01-1R1. As shown in Figure 4 and Supplementary Materials, Table S1, the first transmission experiment was unsuccessful; probably because of inefficient purification of BGDaV1 particles. In the following two experiments, BGDaV1 from two (second transmission experiment) and three (third transmission experiment) donor isolates was successfully transmitted to the virus-free isolate 702.

The stability of successfully transmitted BGDaV1 in each of the newly recovered isolates was tested in three successive subcultures of each isolate. However, successfully transmitted BGDaV1 was not stable in all successive subcultures. In the second experiment, isolates 702-V13 and 702-V101 lost detectable virus in the second and third subcultures, respectively. In the third experiment, isolate 702-V49 lost the virus in the third subculture. It is well known that some mycoviruses are easily lost in culture [46], which could be the case for BGDaV1, possibly after encountering the host’s defence machinery.

### 3.6. Effect of BGDaV1 on B. Cinerea and Its Virulence

**Figure 5:**
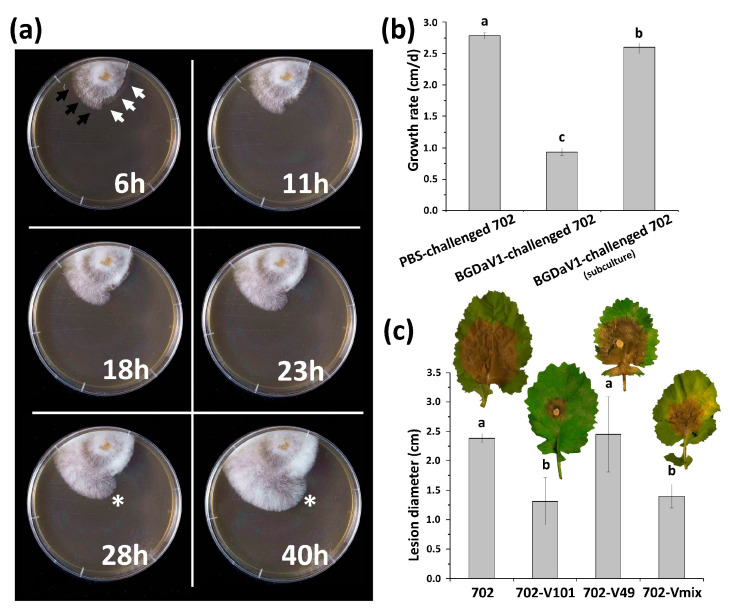
(**a**) A time-lapse comparison of PBS-challenged (indicated by black arrows) and BGDaV1-challenged (indicated by white arrows) colony margins. The PBS-challenged mycelia grew faster than the BGDaV1-challenged side, as indicated by an asterisk; (**b**) Growth rate comparisons between BGDaV1-free and BGDaV1-containing isolates; (**c**) Lesion diameter comparisons between differently treated *B. cinerea* isolates developed on detached leaves of canola. In treatments 702, 702-V101 and 702-V49, mycelial plugs were used to inoculate canola detached leaves. In treatment 702-Vmix, a virus particle mixture purified from fungal isolates 339-49 and 339-101 was applied on canola detached leaves before they were inoculated with mycelial plugs of virus-free isolate 702. Lesion diameter measurements were taken after a 4- to 5-day incubation period of three replicates in each treatment. Different letters indicate significantly different (*p* < 0.050) treatments.

Many mycoviruses have remarkable effects on their pathogenic hosts, including reduced growth rate and virulence. Such virus examples are of special interest as potential biological control agents for fungal diseases [3,45]. SGCV1 affected the host phenotype as well as attenuating its virulence [10]. A time-lapse experiment in which cell-free BGDaV1 purified particles were applied to the growing margins of isolate 702 was performed. Differences in mycelial growth rates along the phosphate-buffered saline (PBS)-challenged and BGDaV1-challenged margins of the 702 colony were observed (Figure 5a). While the PBS-challenged side continued to grow normally, a barrage-like focus of aerial mycelia and restrained substrate mycelia growth was noticed at the virus-challenged side. Significant differences in the growth rate between the PBS- and virus-challenged cultures were detected (Figure 5b). It is worth noting that subculturing the BGDaV1-challenged isolate led to increasing its in vitro growth rate, yet it remained significantly different from the virus-free isolate (Figure 5b). A possible explanation is that the virus was reducing in titre because of host defences during subculturing, as mentioned previously.

To study the effect of BGDaV1 on the pathogenicity of *B. cinerea*, canola detached leaves were inoculated with mycelial plugs of isolates 702, 702-V49 and 702-V101 (produced in the mechanical transmissibility experiment, Supplementary Materials, Table S1). On another occasion, the prophylactic effect of BGDaV1 for *B. cinerea* infection was also evaluated by applying a drop of cell-free BGDaV1 purified particles on canola detached leaves prior to inoculation with mycelial plugs of isolate 702 (treatment: 702-Vmix). The lesion diameters produced by *B. cinerea* isolates 702-V101 and 702-Vmix were significantly less (*p* < 0.050) than those of isolates 702 and 702-V49 (Figure 5c). The increased virulence of isolate 702-V49 could be attributed to the loss of the virus, as previously shown in the mechanical stability experiment (Figure 4).

### 3.7. Accumulation of BGDaV1-derived sRNAs in BGDaV1-Infected Isolates

**Figure 6:**
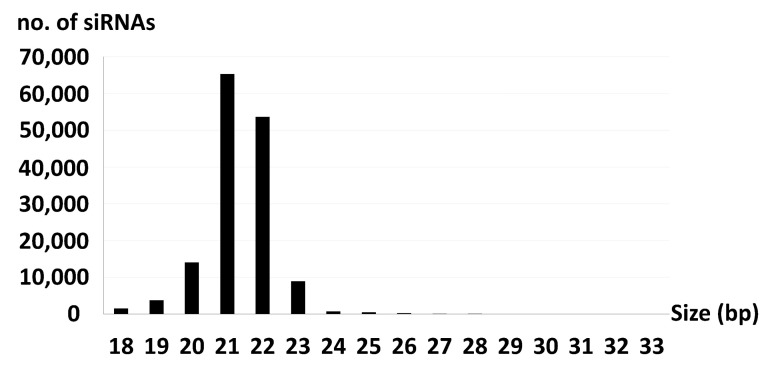
Size distribution of BGDaV1-derived siRNAs.

**Figure 7:**
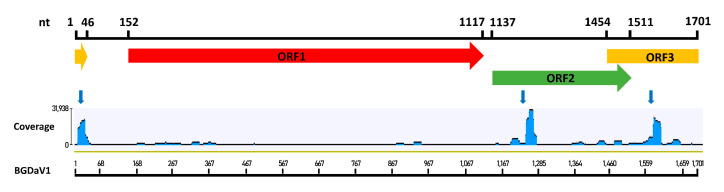
Targeted siRNAs hotspots in BGDaV1 genome (blue arrows).

RNA silencing, also known as quelling in fungi [47,48], acts as an antiviral defence mechanism in some fungi including *B. cinerea* [48,49,50]. A possible explanation for the low titre of BGDaV1 in its natural host was that the virus is targeted by the host defence machinery (RNA silencing and DNA methylation mechanisms). If RNA silencing is active against the virus, then BGDaV1-derived siRNAs would be generated in the botrytis host to target complementary viral RNA for degradation, translational repression, and/or sequence-directed cytosine methylation, resulting in reduced replication and lower virus titres. To test this host antiviral hypothesis for low virus titre, sRNAs analysis was performed. The viral-siRNAs had a size range of 18–33 bp, with 21- and 22-bp long siRNAs being the most numerous, constituting about 44 and 36% of viral-siRNAs, respectively (Figure 6). Three siRNA hotspots were identified as targets for RNA silencing (Figure 7). The first hotspot was located at the stem-loop structure carrying the putative nonanucleotide sequence motif. The remaining two hotspots were located at the start of ORF II and ORF III. These data demonstrate effective antiviral activity in *B. cinerea* akin to efficient gene silencing in *Neurospora crassa* [51], which is responsible for suppressing the replication of several mycovirus discovered recently in this species [52]. On the other hand, this defence mechanism can be overcome [3], as some viruses have counter-defence systems against host RNA silencing, for instance rosellinia necatrix mycoreovirus 3 [53].

### 3.8. Methylation of BGDaV1 Genome

**Figure 8:**
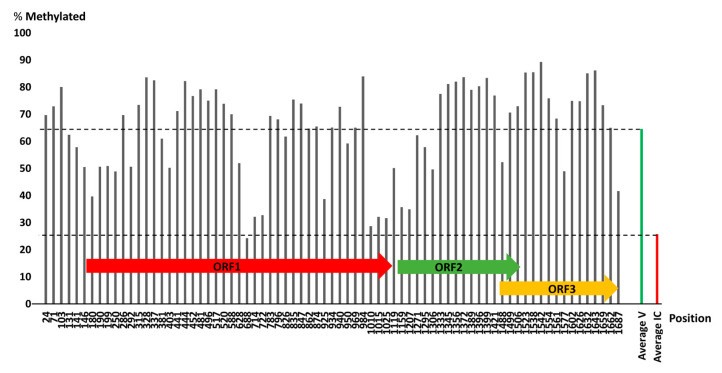
BGDaV1 DNA methylation analysis. Black bars indicate the methylation rate at each CpG position. The green and red bars represent the average cytosine methylation over the entire viral genome and internal control fragment, respectively. The position of each viral ORF is indicated by arrows.

Cytosine-methylation can result in transcription silencing of the viral genome, which results in reduced replication and lower virus titres. Cytosine-methylation is considered as an alternative RNAi-based defence against geminiviruses (a group of plant ssDNA viruses that is closely related to genomoviruses). During infection of plants with geminiviruses, the viral DNA is methylated and host recovery is dependent on hypermethylation of viral DNA [54,55]. Conversely, cytosine methylation may act as a virulence determinant employed by the virus associated with epigenetic status changes of the host fungus [56]. Therefore, a cytosine-methylation profiling experiment was carried out to test the hypothesis that RNA silencing led to an increased methylation of cytosine within the BGDaV1 genome. As shown in Figure 8, the methylation rate over the entire genome of BGDaV1 varied between CpG dinucleotide positions. The average methylation rate over the entire genome was 64.5% (ranging from 24.2 to 89.3%), whereas the average methylation rate over the entire fragment of the *B. cinerea* internal control was 25.7%. The methylation rate was higher than that of the average methylation rate of the *B. cinerea* internal control at almost all CpG positions of the viral genome.

Taken together, the siRNA and cytosine methylation observations provide preliminary evidence that the *B. cinerea* host defence machinery targets the BGDaV1 genome in a sequence-specific manner to down-regulate BGDaV1 RNA accumulation. These fungal host activities probably play a leading role in keeping the virus titre low, resulting in low virus replication capacity, and thus, very low virus incidence in the environment. Such silencing of an infecting virus may result in a long-term mutualistic infection that enables the fungal host to survive along with the viral infection. By contrast, new viral infections of naïve hosts commonly may not present a sufficient antiviral response that negatively affects the virus titre. Hypovirulence in nascent infections was demonstrated in the mechanical transmission experiments whereas detectable BGDaV1 was lost in some subsequent subcultures. The hypovirulence and mechanical transmission attributes of the novel DNA mycovirus BGDaV1, along with its natural biological obsolescence through the host defence machinery, proffer an attractive potential biocontrol activity against its phytopathogenic host, *Botrytis cinerea*.

## 4. Patents

An international patent application from The New Zealand Institute for Plant and Food Research Limited (WO2019/123349) was published under the Patent Cooperation Treaty on 27 June 2019.

## Data Availability

BGDaV1 and BGDaV2 genome sequences are available in GenBank under the accession numbers MK796237 and MZ305104, respectively.

## Supplementary Materials

The following are available online at https://www.mdpi.com/article/10.3390/v13071315/s1, Figure S1: DsRNA profiling of BGDaV1-containing isolates reveals co-infections, Table S1: Mechanical transmission and stability of BGDaV1.

## References

[1] Elad Y., Malathrakis N.E., Dik A.J. (1996). Biological control of *Botrytis*-incited diseases and powdery mildews in greenhouse crops. Crop. Protect..

[2] Williamson B., Tudzynski B., Tudzynski P., van Kan J.A. (2007). *Botrytis cinerea*: The cause of grey mould disease. Mol. Plant. Pathol..

[3] Pearson M.N., Beever R.E., Boine B., Arthur K. (2009). Mycoviruses of filamentous fungi and their relevance to plant pathology. Mol. Plant. Pathol..

[4] Ghabrial S.A. (1998). Origin, adaptation and evolutionary pathways of fungal viruses. Virus Genes.

[5] Xie J., Jiang D. (2014). New insights into mycoviruses and exploration for the biological control of crop fungal diseases. Annu. Rev. Phytopathol..

[6] Bryner S.F., Rigling D. (2012). Hypovirus virulence and vegetative incompatibility in populations of the chestnut blight fungus. Phytopathology.

[7] Yu X., Li B., Fu Y., Jiang D., Ghabrial S.A., Li G., Peng Y., Xie J., Cheng J., Huang J. (2010). A geminivirus-related DNA mycovirus that confers hypovirulence to a plant pathogenic fungus. Proc. Natl. Acad. Sci. USA.

[8] Varsani A., Krupovic M. (2017). Sequence-based taxonomic framework for the classification of uncultured single-stranded DNA viruses of the family *Genomoviridae*. Virus Evol..

[9] Li P., Wang S., Zhang L., Qiu D., Zhou X., Guo L. (2020). A tripartite ssDNA mycovirus from a plant pathogenic fungus is infectious as cloned DNA and purified virions. Sci. Adv..

[10] Yu X., Li B., Fu Y., Xie J., Cheng J., Ghabrial S.A., Li G., Yi X., Jiang D. (2013). Extracellular transmission of a DNA mycovirus and its use as a natural fungicide. Proc. Natl. Acad. Sci. USA.

[11] García-Pedrajas M.D., Cañizares M.C., Sarmiento-Villamil J.L., Jacquat A.G., Dambolena J.S. (2019). Mycoviruses in biological control: From basic research to field implementation. Phytopathology.

[12] Yaegashi H., Shimizu T., Ito T., Kanematsu S. (2016). Differential inductions of RNA silencing among encapsidated double-stranded RNA mycoviruses in the white root rot fungus *Rosellinia Necatrix*. J. Virol..

[13] Rosario K., Duffy S., Breitbart M. (2012). A field guide to eukaryotic circular single-stranded DNA viruses: Insights gained from metagenomics. Arch. Virol..

[14] Goecks J., Nekrutenko A., Taylor J. (2010). Galaxy: A comprehensive approach for supporting accessible, reproducible, and transparent computational research in the life sciences. Genome Biol..

[15] Kearse M., Moir R., Wilson A., Stones-Havas S., Cheung M., Sturrock S., Buxton S., Cooper A., Markowitz S., Duran C. (2012). Geneious Basic: An integrated and extendable desktop software platform for the organization and analysis of sequence data. Bioinformatics.

[16] Edgar R.C. (2004). MUSCLE: Multiple sequence alignment with high accuracy and high throughput. Nucleic Acids Res..

[17] Crooks G.E., Hon G., Chandonia J.M., Brenner S.E. (2004). WebLogo: A sequence logo generator. Genome Res..

[18] Pei J., Grishin N.V. (2014). PROMALS3D: Multiple protein sequence alignment enhanced with evolutionary and three-dimensional structural information. Methods Mol. Biol..

[19] Capella-Gutierrez S., Silla-Martinez J.M., Gabaldon T. (2009). trimAl: A tool for automated alignment trimming in large-scale phylogenetic analyses. Bioinformatics.

[20] Guindon S., Dufayard J.F., Lefort V., Anisimova M., Hordijk W., Gascuel O. (2010). New algorithms and methods to estimate maximum-likelihood phylogenies: Assessing the performance of PhyML 3.0. Syst. Biol..

[21] Stöver B.C., Müller K.F. (2010). TreeGraph 2: Combining and visualizing evidence from different phylogenetic analyses. BMC Bioinform..

[22] Khalifa M.E., Pearson M.N. (2014). Characterisation of a novel hypovirus from *Sclerotinia sclerotiorum* potentially representing a new genus within the *Hypoviridae*. Virology.

[23] White T.J., Bruns T., Lee S., Taylor J., Innis M.A., Gelfand D.H., Sninsky J.J., White T.J. (1990). Amplification and direct sequencing of fungal ribosomal RNA genes for phylogenetics. PCR Protocols: A Guide to Methods and Applications.

[24] Moretti C., Quaglia M., Cerri M., Nicosia D.E., Buonaurio R. (2015). A real-time PCR assay for detection and quantification of *Botrytis cinerea* in *Pelargonium* x *hortorum* plants, and its use for evaluation of plant resistance. Eur. J. Plant Pathol..

[25] Rosario K., Schenck R.O., Harbeitner R.C., Lawler S.N., Breitbart M. (2015). Novel circular single-stranded DNA viruses identified in marine invertebrates reveal high sequence diversity and consistent predicted intrinsic disorder patterns within putative structural proteins. Front. Microbiol..

[26] Hao F., Wu M., Li G. (2021). Characterization of a novel genomovirus in the phytopathogenic fungus *Botrytis cinerea*. Virology.

[27] Ruiz-Padilla A., Rodríguez-Romero J., Gómez-Cid I., Pacifico D., Ayllón M.A. (2021). Novel mycoviruses discovered in the mycovirome of a necrotrophic fungus. Mbio.

[28] Sikorski A., Massaro M., Kraberger S., Young L.M., Smalley D., Martin D.P., Varsani A. (2013). Novel myco-like DNA viruses discovered in the faecal matter of various animals. Virus Res..

[29] Rosario K., Dayaram A., Marinov M., Ware J., Kraberger S., Stainton D., Breitbart M., Varsani A. (2012). Diverse circular ssDNA viruses discovered in dragonflies (Odonata: Epiprocta). J. Gen. Virol..

[30] Dayaram A., Opong A., Jaschke A., Hadfield J., Baschiera M., Dobson R.C., Offei S.K., Shepherd D.N., Martin D.P., Varsani A. (2012). Molecular characterisation of a novel cassava associated circular ssDNA virus. Virus Res..

[31] Ng T.F., Willner D.L., Lim Y.W., Schmieder R., Chau B., Nilsson C., Anthony S., Ruan Y., Rohwer F., Breitbart M. (2011). Broad surveys of DNA viral diversity obtained through viral metagenomics of mosquitoes. PLoS ONE.

[32] van den Brand J.M., van Leeuwen M., Schapendonk C.M., Simon J.H., Haagmans B.L., Osterhaus A.D., Smits S.L. (2012). Metagenomic analysis of the viral flora of pine marten and European badger feces. J. Virol..

[33] Kraberger S., Arguello-Astorga G.R., Greenfield L.G., Galilee C., Law D., Martin D.P., Varsani A. (2015). Characterisation of a diverse range of circular replication-associated protein encoding DNA viruses recovered from a sewage treatment oxidation pond. Infect. Genet. Evol..

[34] Kraberger S., Stainton D., Dayaram A., Zawar-Reza P., Gomez C., Harding J.S., Varsani A. (2013). Discovery of sclerotinia sclerotiorum hypovirulence-associated virus-1 in urban river sediments of Heathcote and Styx rivers in Christchurch city, New Zealand. Genome Announc..

[35] Lamberto I., Gunst K., Muller H., Zur Hausen H., de Villiers E.M. (2014). Mycovirus-like DNA virus sequences from cattle serum and human brain and serum samples from multiple sclerosis patients. Genome Announc..

[36] Uch R., Fournier P.E., Robert C., Blanc-Tailleur C., Galicher V., Barre R., Jordier F., de Micco P., Raoult D., Biagini P. (2015). Divergent gemycircularvirus in HIV-positive blood, France. Emerg. Infect. Dis..

[37] Zawar-Reza P., Arguello-Astorga G.R., Kraberger S., Julian L., Stainton D., Broady P.A., Varsani A. (2014). Diverse small circular single-stranded DNA viruses identified in a freshwater pond on the McMurdo Ice Shelf (Antarctica). Infect. Genet. Evol..

[38] Boevink P., Chu P.W., Keese P. (1995). Sequence of subterranean clover stunt virus DNA: Affinities with the geminiviruses. Virology.

[39] Tisza M.J., Pastrana D.V., Welch N.L., Stewart B., Peretti A., Starrett G.J., Pang Y.-Y.S., Varsani A., Krishnamurthy S.R., Pesavento P.A. (2019). Discovery of several thousand highly diverse circular DNA viruses. BioRxiv.

[40] Dreher T.W., Miller W.A. (2006). Translational control in positive strand RNA plant viruses. Virology.

[41] Miras M., Miller W.A., Truniger V., Aranda M.A. (2017). Non-canonical Translation in Plant RNA Viruses. Front. Plant. Sci..

[42] Khalifa M.E., Varsani A., Ganley A.R.D., Pearson M.N. (2016). Comparison of Illumina de novo assembled and Sanger sequenced viral genomes: A case study for RNA viruses recovered from the plant pathogenic fungus *Sclerotinia sclerotiorum*. Virus Res..

[43] Ikeda K., Nakamura H., Arakawa M., Matsumoto N. (2004). Diversity and vertical transmission of double-stranded RNA elements in root rot pathogens of trees, *Helicobasidium mompa* and *Rosellinia necatrix*. Mycol. Res..

[44] Tuomivirta T.T., Hantula J. (2005). Three unrelated viruses occur in a single isolate of *Gremmeniella abietina* var. abietina type A. Virus Res..

[45] Ghabrial S.A., Caston J.R., Jiang D., Nibert M.L., Suzuki N. (2015). 50-plus years of fungal viruses. Virology.

[46] Bao X., Roossinck M.J. (2013). Multiplexed interactions: Viruses of endophytic fungi. Adv. Virus Res..

[47] Romano N., Macino G. (1992). Quelling: Transient inactivation of gene expression in *Neurospora crassa* by transformation with homologous sequences. Mol. Microbiol..

[48] Torres-Martínez S., Ruiz-Vázquez R.M. (2017). The RNAi universe in fungi: A varied landscape of small RNAs and biological functions. Annu. Rev. Microbiol..

[49] Segers G.C., Zhang X., Deng F., Sun Q., Nuss D.L. (2007). Evidence that RNA silencing functions as an antiviral defence mechanism in fungi. Proc. Natl. Acad. Sci. USA.

[50] Patel R.M., van Kan J.A., Bailey A.M., Foster G.D. (2008). RNA-mediated gene silencing of superoxide dismutase (*bcsod1*) in *Botrytis cinerea*. Phytopathology.

[51] Cogoni C., Macino G. (1999). Homology-dependent gene silencing in plants and fungi: A number of variations on the same theme. Curr. Opin. Microbiol..

[52] Honda S., Eusebio-Cope A., Miyashita S., Yokoyama A., Aulia A., Shahi S., Kondo H., Suzuki N. (2020). Establishment of *Neurospora crassa* as a model organism for fungal virology. Nat. Commun..

[53] Yaegashi H., Yoshikawa N., Ito T., Kanematsu S. (2013). A mycoreovirus suppresses RNA silencing in the white root rot fungus, *Rosellinia necatrix*. Virology.

[54] Paprotka T., Deuschle K., Metzler V., Jeske H. (2011). Conformation-selective methylation of geminivirus DNA. J. Virol..

[55] Rodriguez-Negrete E.A., Carrillo-Tripp J., Rivera-Bustamante R.F. (2009). RNA silencing against geminivirus: Complementary action of posttranscriptional gene silencing and transcriptional gene silencing in host recovery. J. Virol..

[56] Nuskern L., Jezic M., Liber Z., Mlinarec J., Curkovic-Perica M. (2018). *Cryphonectria hypovirus 1*-induced epigenetic changes in infected phytopathogenic fungus *Cryphonectria parasitica*. Microb. Ecol..

