# Supplementary material for: A Mechanically Transmitted DNA Mycovirus Is Targeted by the Defence Machinery of Its Host, Botrytis cinerea
Source: Viruses. 2021 Jul 7;13(7):1315. doi: 10.3390/v13071315 (PMC8309985; doi:10.3390/v13071315)
Supplement: Supplementary file 1 [file viruses-13-01315-s001.zip › viruses-1271976-supplementary.pdf]

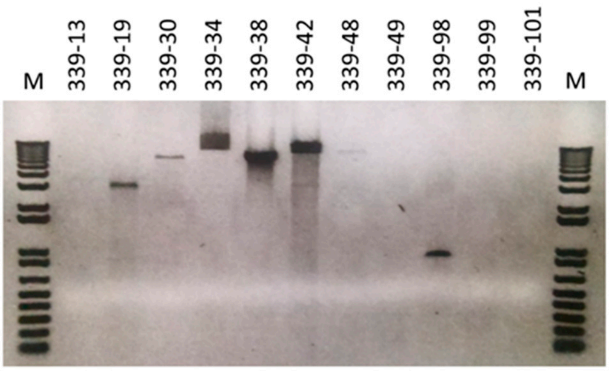

Figure S1. DsRNA profiling of BGDaV1-containing isolates reveals co-infections. M: 1kb Plus DNA molecular weight marker (Invitrogen).

Supplementary Table S1. Mechanical transmission and stability of BGDaV1. Presence (+) or absence (-) of BGDaV1 in different sub-cultures of newly developed progeny as detected by PCR.

	Experiment 1			Experiment 2			Experiment 3		
**Virus donor**	339-13	339-49	339-101	339-13	339-49	339-101	339-13	339-49	339-101
**Sub-culture 1**	No transmission			+	+	+	-	+[1]	+[1]
**Sub-culture 2**				-	+	+	-	+	+
**Sub-culture 3**				-	+	-	-	-	+

[1] isolates used for growth rate and virulence assessment.
